# Gasdermin family: a promising therapeutic target for asthma

**DOI:** 10.3389/fmed.2026.1763400

**Published:** 2026-04-16

**Authors:** Chaoping Wang, Xuelin Zhang, Fang Cheng, Haiying Huang, Xiao Li

**Affiliations:** 1 Henan University of Chinese Medicine, Zhengzhou, China; 2 The Third Affiliated Hospital of Henan University of Chinese Medicine, Zhengzhou, China

**Keywords:** asthma, Gasdermin family, inflammation, pyroptosis, therapeutic target

## Abstract

As key regulators of inflammation and cell death, gasdermins (GSDMs) play a pivotal role in the pathogenesis of asthma. Through regulation via single nucleotide polymorphisms (SNPs), mediation of pyroptosis, and modulation of signaling pathways such as the MAVS-TBK1 pathway, GSDMs promote mucus hypersecretion, airway epithelial barrier dysfunction, and persistent inflammation, thereby exacerbating airway inflammation, airway remodeling, and pulmonary fibrosis. With the continuous expansion and deepening of research, GSDM-targeted inhibitors show great promise for precision therapy. Meanwhile, GSDM expression levels and specific SNPs may serve as potential biomarkers for the diagnosis and prognosis of asthma, providing novel strategies for asthma management.

## Introduction

Asthma is regarded as one of the common respiratory diseases. According to the findings of the Global Burden of Disease Study 2021, the age-standardized incidence rate of asthma globally was 736 per 100,000 population in 1990, while the age-standardized prevalence rate was 5,568 per 100,000 population. By 2021, these indicators had decreased to 516 and 3,340 per 100,000 population, respectively. Both the incidence and prevalence of asthma showed a continuous downward trend from 1990 to 2021, yet asthma still ranked among the leading chronic respiratory diseases in terms of global disease burden. Based on predictions from the Bayesian age-period-cohort model, the global age-standardized prevalence rate of asthma is projected to be 2,566 per 100,000 population by 2050, and the age-standardized incidence rate will remain relatively stable with a slight increase until 2050, reaching approximately 520 per 100,000 population by 2050. Meanwhile, the global number of asthma cases will rise from 260 million in 2021 to 275 million in 2050. This suggests that asthma will continue to pose a major public health burden for a long time (1, 2). Currently, the combination of inhaled corticosteroids and long-acting *β*₂-receptor agonists (ICS/LABA) remains the first-line treatment option for asthma. However, studies have shown that even with standardized treatment, 28–80% of patients still have poorly controlled asthma (3). Meanwhile, asthma patients worldwide also face a heavy economic burden. For example, the median annual asthma-related medical expenses for patients in the United States reach $3,544, and the annual medical expenditure for patients in China is as high as 23,037 CNY (4, 5). Therefore, there is an urgent need to continuously explore new targeted therapies and comprehensive management strategies for asthma prevention and control to further improve patients’ prognosis and enhance their quality of life.

**Figure 1:**
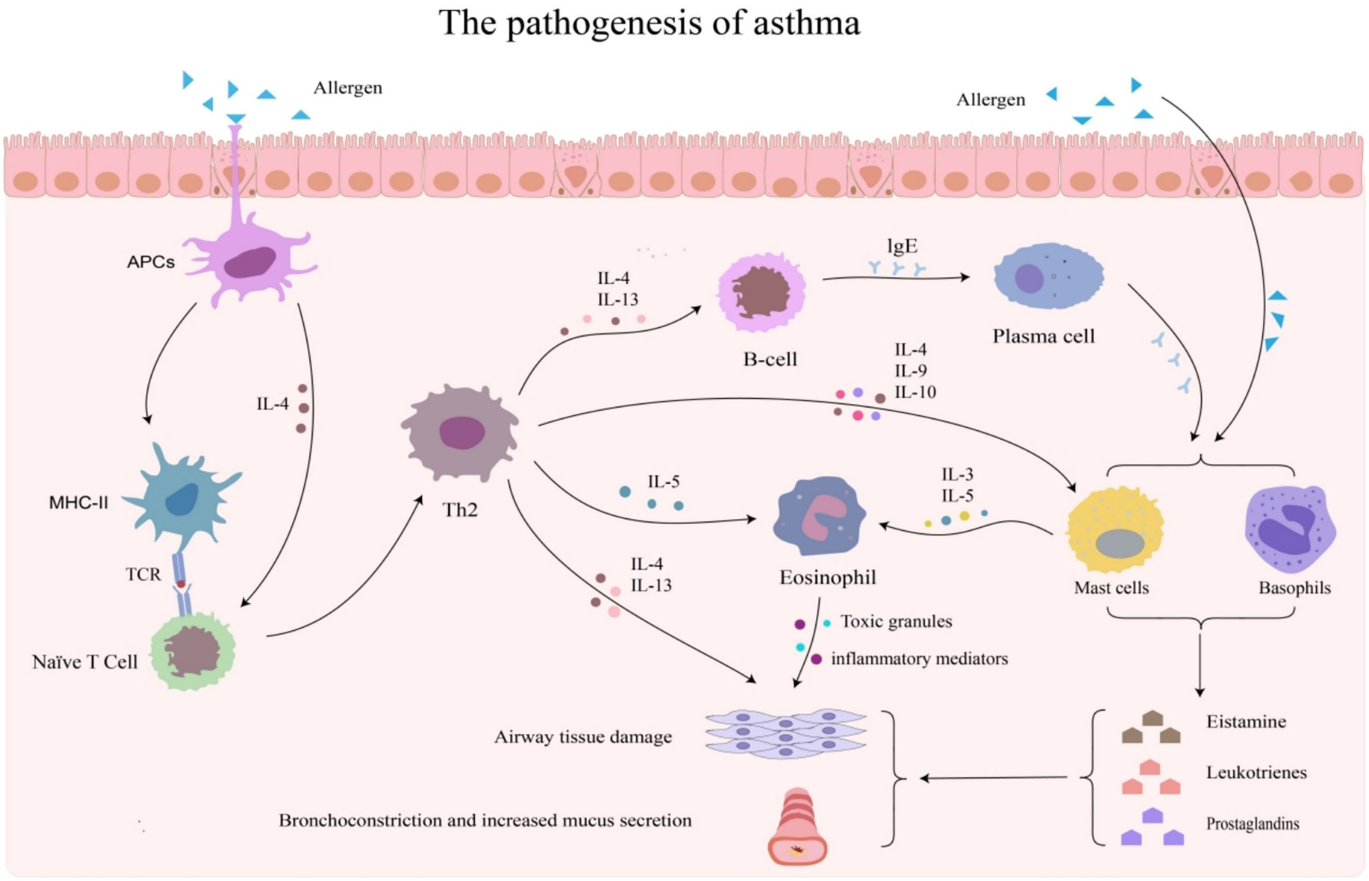
The pathogenesis of asthma involves several key processes: Initiation: allergens enter the body, activate antigen-presenting cells (APCs), and initiate an immune response. Differentiation: APCs activate naïve T cells, causing them to differentiate into Th2 cells. Signal transduction: Th2 cells secrete cytokines, which encourage B cells to generate IgE. Sensitization: mast cells and basophils become sensitized when IgE binds to their receptors. Triggering: when the allergen is encountered again, IgE binds with it, causing mast cells and basophils to release their granules. Release of inflammatory mediators: mast cells and basophils release inflammatory mediators, leading to airway constriction and inflammation. Infiltration of inflammatory cells: chemokines attract inflammatory cells (such as eosinophils) to accumulate in the airways. Chronic inflammation and remodeling: eosinophils release toxic granules and inflammatory mediators, resulting in airway remodeling and persistent chronic inflammation.

Asthma is closely related to both genetic factors and environmental influence (6). It can be divided into “Type-2-high” asthma and “Type-2-low” asthma. “Type-2-high” asthma refers to allergic and eosinophilic asthma, while “Type-2-low” asthma encompasses neutrophilic, paucigranulocytic, and obesity asthma. Clinical data show that around half of the patients with mild to moderate asthma, as well as most patients with severe asthma, are driven by Th2-dependent inflammation (7) (Figure 1). Related research is constantly being supplemented. Here, we only list some of the immune mechanisms. The HIF2α-GATA3 (Hypoxia-inducible factor 2α-GATA-binding protein 3) circuit promotes pathogenic Th2 cell differentiation by regulating the transcription of inositol polyphosphate multikinase (8). Impaired regulatory T (Treg) cells weaken the inhibition of Th2 cells, further amplifying Th2-mediated inflammatory responses (9). Airway epithelial cells release alarmins, such as IL-33, TSLP, that can activate type 2 innate lymphoid cells to secrete IL-5 and IL-13, which exert a synergistic effect with Th2 cytokines (10). Meanwhile, elevated serum levels of complement components (C3, C3a, C4a, etc.) in patients with asthma are connected to disease severity, indirectly exacerbating Th2-dominated inflammatory processes (11, 12).

The core pathological mechanism triggering asthma is an imbalance in Th2 inflammation, which is mainly driven by key cytokines, such as IL-4, IL-5, IL-13 (13). On the one hand, these cytokines can alter airway structure, promote mucus secretion, and exacerbate smooth muscle hypertrophy, leading to cell production and airflow limitation (14). On the other hand, they act on various types of cells to produce inflammatory mediators, trigger airway inflammation, and ultimately induce asthma (15). Recent research has found that Gasdermin Family (GSDMs), as membrane-perforating proteins, may disrupt tissue integrity by inducing pyroptosis and stimulating the expression of bioactive interleukin (including IL-33, IL-18) from cells (13). The released IL-33 directly acts on basophilic granulocytes through the ST2 signaling pathway, inducing the production of IL-4 and upregulating the recruitment of integrin-dependent Th2 cells to the lung tissue (16). IL-18 can induce Th2 and Th17 responses, secreting IL-4, IL-5, IL-13, and IL-17A, and continuously maintain chronic airway inflammation (17). This paper reviews the mechanism of GSDMs in asthma. GSDMs participate in the occurrence and development of asthma by inducing key pathological processes, including airway inflammation, excessive mucus secretion, epithelial barrier disruption, and airway remodeling. By exploring the key signaling pathways through which GSDMs regulate asthma and performing targeted interventions on its core molecules, it can provide a theoretical reference for identifying potential therapeutic targets for asthma.

## The physiological functions of the Gasdermin family in asthma

In 2000, Japanese scientists isolated and identified a novel *mouse* gene named Gasdermin, which was also found to be present in the *human* genome (18). At present, we find that GSDMs are key participants in inflammatory diseases and the execution of pyroptosis, playing a significant role in the pathophysiological processes of asthma (19, 20). The Gasdermin family in *human* genes includes six members: Gasdermin A (GSDMA), Gasdermin B (GSDMB), Gasdermin C (GSDMC), Gasdermin D (GSDMD), Gasdermin E (DFNA5), and Gasdermin F (pejvakin, PJVK) (21). They are distributed at different chromosomal locations and correspond to different diseases. GSDMA and GSDMB are situated on chromosome 17q21, which is frequently associated with asthma susceptibility (22); GSDMC and GSDMD map to the 8q24 region, which coincides with a genomic locus linked to cancer risk (23, 24); GSDME maps to chromosome 7p15.3, while PJVK resides on chromosome 2q31.2, both of which are related to deafness (25, 26).

GSDMs can trigger cell death and release pro-inflammatory cytokines, playing a crucial regulatory role in the inflammatory response (27). Structurally, most GSDM proteins (except PJVK) have a C-terminal domain (CTD), an N-terminal domain (NTD) effector region, and a flexible connecting linker. The full-length GSDM protein has a self-inhibitory feature, as the basic β1–β2 loop and α1 helix of the NTD are masked by the acidic region of the CTD, forming a closed structure with no membrane-binding activity (28). When the upstream protease is activated, it can perform a specific cleavage at the cleavage site in the linker region. The CTD dissociates from the NTD, and the basic region of NTD forms an electrostatic interaction with the acidic phospholipid head group of the membrane (29). The *β*-hairpin conformation rearranges and inserts into the lipid bilayer. Subsequently, they assemble into a ring-shaped oligomer and form a membrane pore. In this process, the presence of the CTD is essential. It prevents spontaneous cell death through intramolecular inhibition. The GSDM - CTD (excluding PJVK) constrain the NTD via intramolecular inhibition, preventing its spontaneous assembly into membrane pores, and thereby avoiding diseases induced by excessive activation of pyroptosis. Only upon activation by upstream signaling does dissociation occur between CTD and NTD, allowing the NTD to expose its membrane-binding region and form regulated membrane pores, which precisely trigger pyroptosis as an immune defense mechanism (30). The inner diameters of most protein pores range from 16 to 33 nanometers, and are composed of 26–54 protomers (31). Pyroptosis is a lytic pro-inflammatory mechanism used to regulate cell death, marked by cell rupture, and the release of pro-inflammatory cytokines, including IL-1β, IL-18 and high mobility group protein B1 (HMGB1) (32). These factors can promote the persistence and exacerbation of pulmonary inflammation.

NOD-like receptor protein 3 (NLRP3) inflammasome, a multi-protein complex consisting of NLRP3, apoptosis-associated speck-like protein (ASC), and pro-caspase-1, is a core upstream driver of pyroptosis and plays an important role in immune response to pathogen stimulation (33). Meanwhile, the NLRP3 inflammasome is the core upstream driving factor that induces pyroptosis in cells. First, Toll-like receptors are activated by pathogen-associated molecular patterns or damage-associated molecular patterns, activating the NF-κB pathway to promote the transcriptional expression of NLRP3 and pro-caspase-1 (34). Subsequently, NLRP3 binds to ASC via its pyrin domain, and ASC recruits pro-caspase-1 to form a functional NLRP3 inflammasome complex (35). The complex then induces self-cleavage of pro-caspase-1 into mature caspase-1, which specifically cleaves GSDMD at the Asp276 site to release its NTD (33, 36). Finally, GSDMD-NTD is localized to the cell membrane via palmitoylation, oligomerizes to form transmembrane pores, mediates the release of pro-inflammatory cytokines (e.g., IL-1β, IL-18), and induces cell swelling and lysis to complete pyroptosis, forming the complete molecular pathway of NLRP3-mediated pyroptosis (35, 37).

The GSDMs regulate the inflammatory response and airway function in asthma through various mechanisms: a. Pyroptosis, the core mechanism of pyroptosis involves the activation of the GSDMs-NTD, which form membrane pores. These membrane pores in turn release inflammatory factors and regulate airway inflammation (38). b. SNPs, Genome-wide Association Study (GWAS) shows that SNPs in GSDMA and GSDMB are connected to the risk, severity, and exacerbations of asthma. These SNPs can influence antiviral pathways and the expression of GSDMs (39). c. Signaling pathways, GSDMB, for example, can activate the mitochondrial antiviral signaling protein (MAVS)-TANK binding kinase 1 (TBK1) signaling pathway, enhancing the expression of interferon-stimulated genes (ISGs), 5-lipoxygenase (5-LO) and transforming growth factor-β1 (TGF-β1), exacerbating airway inflammation, airway remodeling and airway hyperresponsiveness (AHR) (40). Many studies have confirmed that GSDMs are associated with asthma and have become a promising target for asthma treatment (Table 1).

**Table 1:** The GSDM family: expression, functions, and implications for disease.

**Gasdermins**	**Gene and chromosomal location**	**Pyroptosis-inducing activity**	**Tissue expression**	**Associated disease**
*Human*: GSDMA *Mouse*: Gadma1 Gadma2 Gadma3	*Human*: chr17q21 *Mouse*: chr11D	Yes	gastrointestinal tract T lymphocytes mammary glands bladder skin	Asthma (98) Systemic sclerosis (99) Atopic dermatitis (100)
*Human*: GSDMB *Mouse*: Not	*Human*: chr17q21	Yes	Gastrointestinal tract endocrine tissue Airway epithelium Endocrine Brain Lung	Asthma (101) Allergic rhinitis (22) cancer (102) Inflammatory bowel disease (102)
Human: GSDMC *Mouse*: Gsdmc1 Gsdmc2 Gsdmc3 Gsdmc4	*Human*: chr8q24.2 *Mouse*: chr15D1	Yes	Cerebral cortex Skin Trachea Spleen Digestive tract Vagina Bladder Upper airway	Pancreatic cancer (103) Atopic dermatitis (104) Allergic rhinitis (105)
*Human*: GSDMD *Mouse*: Gsdmd	*Human*: chr8q24.3 *Mouse*: chr15D3	Yes	Most human organs and tissues, along with various leukocyte types	Asthma (106) Atherosclerosis (107) Type 2 diabetes mellitus (108) Atopic dermatitis (109)
*Human*: GSDME/DFNA5 *Mouse*: Gsdme	*Human*: chr7p15.3 *Mouse*: chr6B2.3	Yes	Endocrine tissue Gastrointestinal tract Muscle tissue Brain Endometrium and placenta	Deafness (84) Tumor (84) Atopic dermatitis (110)
*Human*: GSDMF/PJVK *Mouse*: Pjvk	*Human*: chr2q31.2 *Mouse*: chr2.3	No	Ear	Deafness (26)

## Gasdermin A in asthma

At the genetic expression level, GWAS has confirmed that the GSDMA gene located at 17q21 is closely related to the risk of asthma onset (41). GSDMA contains four common coding variants related to asthma susceptibility (rs7212944, rs56030650, rs7212938, and rs3894194), most of which have also been identified by other GWASs reports on GSDMA and asthma (22). Another study confirmed through *in vitro* cell experiments that the asthma-risk SNPs located at 17q21 are significantly associated with the upregulation of mRNA transcription levels and protein expression of the GSDMA gene in neonatal cord blood mononuclear cells (CBMCs) after stimulation with the major allergen Der p1 from Dermatophagoides pteronyssinus (42). These findings suggest that genetic variations in 17q21 may participate in the regulation of asthma susceptibility and disease progression by targeting and modulating the expression of GSDMA, thereby enhancing the sensitivity of early immune cells (such as CBMCs) to dust mite allergens.

In addition, the protease virulence factor of SpeB released by *Streptococcus pyogenes* can specifically cleave GSDMA at the Gln246 site, generating a bioactive N-terminal region (43, 44). This N-terminal fragment can penetrates the cell membrane to form pores, inducing pyroptosis and releasing pro-inflammatory factors (44). However, this process has mainly been observed in the highly-expressing skin (45). Current research has verified that GSDMA is found in skin tissues, airway epithelial cells, and lung adenocarcinoma, but there are significant differences in its expression patterns: the skin is the primary physiological expression site of GSDMA, while the basal expression level in airway tissues is relatively low (46–48). Currently, there is no investigation into how GSDMA-mediated pyroptosis affects asthma. However, considering that the pathophysiological processes of asthma mainly involve airway inflammation and epithelial dysfunction, GSDMA may affect asthma by mediating airway cell pyroptosis and cytokine release upon activation; but this remains to be investigated.

## Gasdermin B in asthma

**Figure 2:**
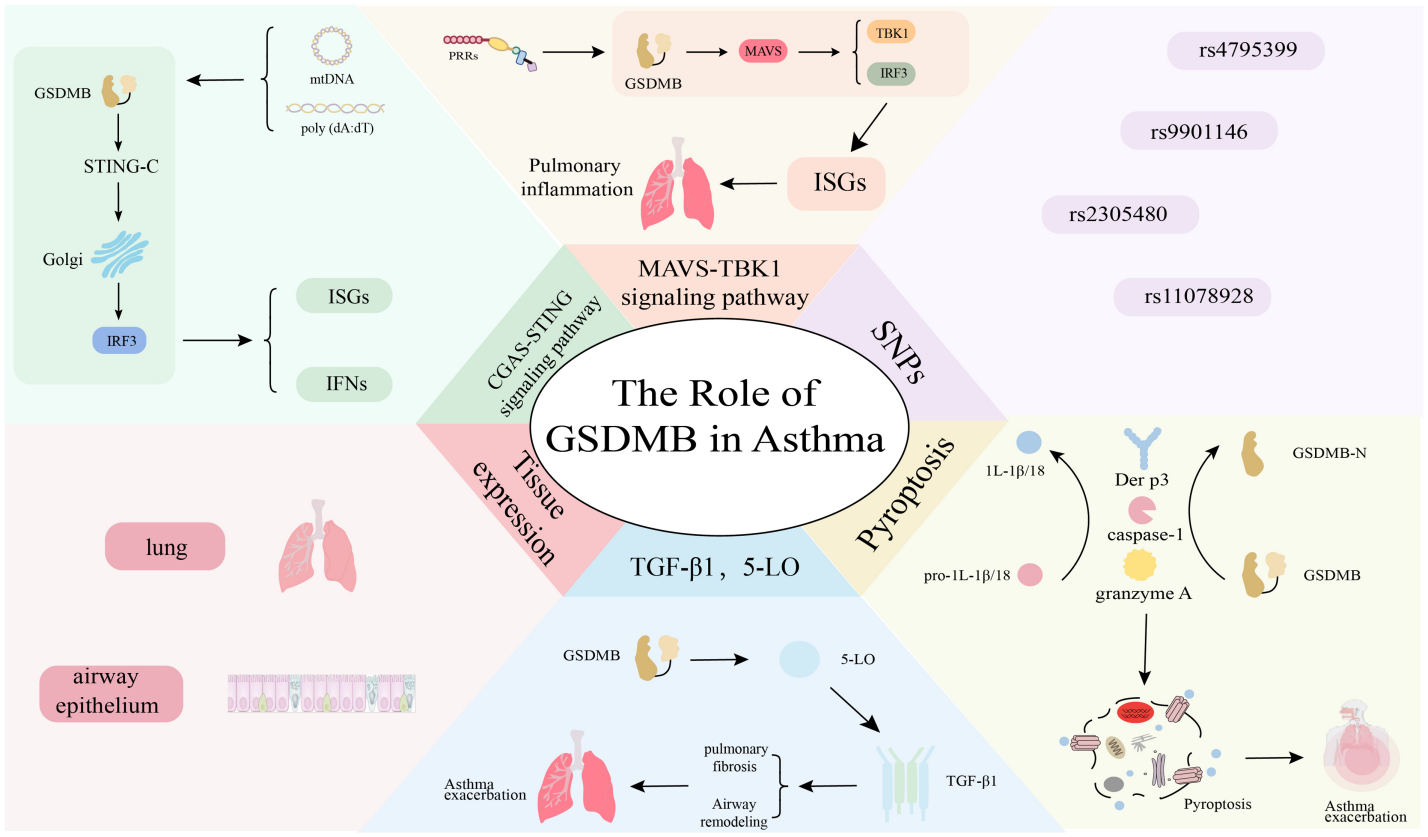
GSDMB can affect the onset of asthma in the following aspects: (1) ISGs: GSDMB enhances the expression of ISGs by activating the MAVS-TBK1 and cGAS-STING signaling pathways, thereby enhancing antiviral and inflammatory responses. (2) SNPs: research has demonstrated a significant link between *GSDMB* gene polymorphisms and asthma susceptibility, severity, exacerbation, and immune responses (such as IgE levels). (3) Pyroptosis: GSDMB contributes to asthma development by triggering pyroptosis. Once caspase-1 cleaves the N-terminal domain of GSDMB, it forms pores in the cell membrane, releasing inflammatory factors that then initiate an inflammatory response. (4) TGF-β1 and 5-LO: the overexpression of GSDMB enhances the levels of 5-LO and TGF-β1, leading to airway remodeling and pulmonary fibrosis. (5) Tissue expression: GSDMB shows high expression in the lungs and airway epithelial cells of asthma patients, correlating with asthma severity, the likelihood of exacerbations, and involvement in the antiviral pathway.

GSDMB is a key gene linked to asthma susceptibility found in the 17q21 region (49) (Figure 2). In recent years, accumulating research has increasingly solidified evidence for the link between GSDMB and asthma (50). For instance, a meta-analysis on childhood-onset asthma in white individuals of European ancestry demonstrated that the rs4795399 locus within GSDMB exhibited the most significant genome-wide association signal (*p* = 1 × 10^−257^), providing compelling support for its robust link to childhood asthma (51). Subsequently, Ronald et al. identified two functional coding variants in GSDMB (rs2305480 and rs11078928) linked to a lower risk of asthma, validated in two independent cohorts: the GERA cohort (OR = 0.92; *p* = 1.01 × 10^−6^) and EVE cohort (OR = 0.85; joint PEVE = 1.31 × 10^−13^) (52). It is worth noting that the major T allele of the rs11078928 SNP increases the risk of asthma, while the C allele can lead to decreased pyroptosis activity of GSDMB, potentially resulting in a reduced occurrence of asthma (53). Another study has confirmed that the minor genotype of rs7216389 can significantly reduce the level of IgE in the serum of children with asthma, thereby lowering the risk of asthma attacks and disease exacerbations (54). And allele frequency analysis revealed that the minor allele of rs2305480 was significantly more prevalent in controls than in cases (U. S.: 0.45 vs. 0.39; EVE: 0.32 vs. 0.27), indicating that the protective effect of this variant is generalizable across populations (52). Additionally, Sarnowski et al. focused on the phenotype of asthma onset age and found that the intergenic rs9901146 locus between GSDMB and zona pellucida-binding protein 2 showed the strongest link to this trait, offering a new perspective on GSDMB’s role in asthma progression (55). A genetic link between GSDMB and asthma has been clearly established, but the specific mechanism remains unclear. A study has proposed that local CpG DNA methylation significantly influences the function of GSDMB-related expression quantitative trait loci, which can regulate GSDMB expression and epigenetic modification, affecting its association with asthma-related phenotypes, although some mechanisms are unclear (56). However, this study has limitations, including a narrow experimental scope, incomplete exploration of regulatory mechanisms, and reliance solely on correlation analysis without dynamic or interventional evidence, thus requiring further verification.

Many studies have confirmed that GSDMB also participates in the pathogenesis of asthma via non-genetic mechanism pathways. Liu et al. demonstrated that GSDMB functions as a critical upstream regulator of the MAVS-TBK1 signaling pathway. Independent of canonical RNA sensors, GSDMB acts as a novel RNA sensor that directly interacts with biotin-labeled poly(I:C) through its amino acid residues 83–274, leading to enhanced activation of the MAVS-TBK1 cascade. This mechanism potently induces the expression of interferon-stimulated genes (ISGs) and exacerbates airway inflammation (40). Similarly, in airway epithelial cells, GSDMB actively boosts cGAS-STING pathway activation upon stimulation with Mitochondrial DNA and poly (dA:dT), promotes the induction of ISGs, and is associated with asthma (57). The full-length GSDMB can be cleaved by caspase-1 at the D236 position and be cut at the K244 position by the house dust mite protease Der p3 or granzyme A, releasing the active GSDMB-NT which induces cell pyroptosis and releases inflammatory factors. Studies have confirmed that both caspase-1 and granzyme A are elevated in asthma (58, 59). The presence of GSDMB in the airway epithelium and lung leads to pyroptosis, IFN release, impairment of airway epithelial cell integrity and disruption of the airway mucosal barrier (60, 61). This makes it easier for allergens, viruses, and other agents to invade airway tissues, triggering the initial inflammatory response and worsening the wheezing caused by viral infections, thereby laying hidden dangers for the onset and exacerbation of asthma (61). Functional studies have shown that GSDMB directly upregulates 5-LO and TGF-β1 through transcriptional activation. These two molecules play a crucial role in airway remodeling and AHR, thereby participate in the pathological process of asthma (62). In this process, the 5-LO activity induced by GSDMB enhances TGF-β1 expression in bronchial epithelial cells. TGF-β1 can promote the growth and movement of airway smooth muscle cells, cause structural changes in the airway that result in airway remodeling, and stimulate fibroblast growth and extracellular matrix formation, leading to pulmonary fibrosis (62). Furthermore, although existing studies have shown that this process does not directly involve lung inflammation, TGF-β1, as a key factor in inflammation regulation, can activate the transcription factor NF-κB to upregulate the expression of pro-inflammatory cytokines, thereby inducing airway inflammation (63). The mechanism of its inflammatory regulation is complex and still requires in-depth exploration. This may provide new ideas for the prevention and control of airway remodeling and inflammatory responses in respiratory system diseases.

## Gasdermin D in asthma

**Figure 3:**
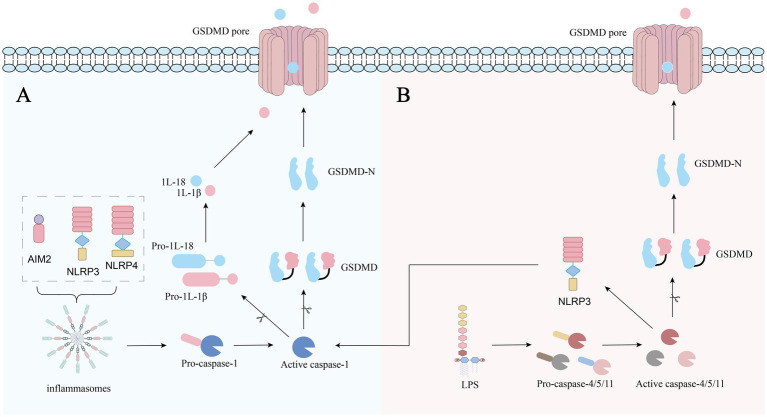
**(A)** The canonical pathway is the “caspase-1-dependent pathway.” In this pathway, the main process involves the activation of inflammasomes (including NLRP3, NLRC4, AIM2, etc.) (95). These inflammasomes are activated after stimulated by pathogens or toxins and then activate caspase-1, which cleaves GSDMD to produce the GSDMD-NT domain. This domain forms pores in the cell membranes, causing its rupture and resulting in pyroptosis (95). Moreover, caspase-1 cleaves the pro-IL-1β and pro-IL-18, forming IL-1β and IL-18, which can boost the inflammatory response (96). **(B)** The main feature of the non-canonical pathway (caspase-4/5/11-dependent pathway) is that lipopolysaccharide (LPS) activates caspase-4/5/11 without the participation of inflammasomes and subsequently cleaves GSDMD to generate the GSDMD-NT active domain, causing cell membrane perforation and pyroptosis (68). In certain situations, caspase-4/5/11 might promote the activation of caspase-1 by activating the NLRP3 inflammasome, thereby amplifying the inflammatory response (97).

The impact of GSDMD in asthma is mainly achieved through pyroptosis, a process in which cells expand until the membrane bursts, releasing a large amount of inflammatory factors, which subsequently trigger a strong inflammatory response (64). Pyroptosis operates through two main pathways, with GSDMD serving as the common key execution factor (65) (Figure 3). In the process of pyroptosis, GSDMD-NT active fragment, which is approximately 32 amino acids, oligomerizes on the cell membrane to form pores measuring 10–14 nm in diameter. These pores keep increasing, eventually causing cell rupture and pyroptosis, releasing all the contents within the cell and triggering an inflammatory reaction (66). Additionally, activated caspase-1 cleaves pro-IL-1β and pro-IL-18, while caspase-4 cleaves pro-IL-18, forming active IL-1β and IL-18 (67, 68). Due to their smaller diameters compared to the oligomerized membrane pores, these cytokines are able to pass through the membrane pores, thereby amplifying the inflammation (69). This dual activation drives a positive feedback loop: GSDMD creates membrane pores, releasing IL-1β, IL-18, and damage-associated molecular patterns such as HMGB1 (70). IL-1β and IL-18 induce a precisely coordinated inflammatory reaction to initially establish an inflammatory microenvironment (71), while HMGB1 promotes airway remodeling by regulating inflammation and activating lung fibroblasts (72). Ultimately, they can trigger a persistent inflammatory response, promote swelling of airway mucosa and increase of mucus secretions, and induce AHR and airway remodeling. Meanwhile, pyroptosis-mediated cell death further amplifies the inflammatory cascade, and sustained inflammation ultimately induces AHR and structural changes associated with airway remodeling, exacerbating the clinical symptoms of asthma (60). Increasing research results suggest a fundamental relationship between asthma and pyroptosis, although its molecular alterations contributing to the disease are unkown.

Pyroptosis is the most important pathway for GSDMD to involve the process of asthma. Besides, there are other pathways which can cause the symptoms of asthma. Another study found that GSDMD can promote airway inflammation and remodeling, possibly related to the Th17 inflammatory response and M2 macrophage polarization (73, 74). Th17 cells, a type of CD4^+^ T cell, mainly secrete IL-17, which drives neutrophil recruitment and amplifies inflammatory responses, leading to airway inflammation, airway remodeling, and airway hyperresponsiveness (75). In addition, after allergens activate epithelial cells, GSDMD undergoes caspase-1/11-independent cleavage, generating an amino-terminal p40 fragment. The GSDMD p40 fragment forms transmembrane pores in the cell membrane via self-oligomerization, allowing interleukin-33 to be released into the extracellular space (76). IL-33 regulates allergic asthma in a manner that depends on age. In childhood, it activates ILC2s and mast cells and promotes Type 2 cytokines, exacerbating asthmatic phenotypes. In adults, its pro-asthmatic effects weaken, reducing Type 2 inflammation via Tregs/IL-10 or involving non-Type 2 asthma. In the elderly, it drives less Type 2 inflammation but may contribute to chronic airway remodeling (77).

## Other GSDMs in asthma

Currently, research on the functions of other GSDMs in asthma is relatively limited. This article focuses on sorting out and summarizing existing research content, presenting the established research foundation and key clues in this field. GSDMC was originally detected in the upper gastrointestinal tract (78). Currently, it has been found to be associated with lung diseases. In patients with chronic obstructive pulmonary disease (COPD), GSDMC is notably upregulated in *human* lung epithelial cells, and its expression level changes significantly in response to external stimuli such as LPS and cigarette smoke extract, although the N-terminal fragment of GSDMC in bronchoalveolar lavage fluid was not directly detected (47). As two common chronic progressive lung diseases, asthma and COPD can exist together, a condition known as asthma-COPD overlap (ACO) (79). Approximately 20% of COPD patients and about 14% of asthma patients are diagnosed as ACO (80). This indicates that part of the pathological basis of COPD and asthma may be similar, and the airway inflammation and lung damage caused by COPD may have an impact on asthma. Moreover, there was an inverse correlation between DNA methylation and GSDMC expression. High methylation values were associated with better survival rates in lung adenocarcinoma, while stronger expression of GSDMC was associated with a poor prognosis (81). DNA methylation is an epigenetic modification mechanism. Its core characteristic is achieved by adding a methyl group (-CH₃) to nitrogenous bases. This process mainly occurs at CpG sites, which are dinucleotide sequences where cytosine (C) is directly linked to guanine (G) (CpG dinucleotides) (82). In recent years, it was confirmed that differential methylation can promote lgE sensitization, influence lung function, and affect the severity and occurrence of asthma (83). Additionally, caspase-8 cleaves and activates GSDMC, triggering pyroptosis, which may release inflammatory factors and participate in inflammatory airway diseases such as asthma (19). These findings suggest that GSDMC may damage the lungs and reduce lung function, thereby affecting asthma. However, further verification is required to confirm the authenticity and reliability of GSDMC as a promising therapeutic target for asthma.

GSDME was originally recognized as a key gene linked to progressive hearing loss (84). Recently, most research has focused on GSDME-mediated pyroptosis in anti-tumor effects. GSDME becomes active after cleaved by caspase-3 or granzyme B; it releases the N-terminal domain, which mediates the tumor cell lysis and triggers an anti-tumor inflammatory response (85, 86). Like GSDMD, GSDME is crucial in pyroptosis, and it can damage the lung (87). A decrease in lung function may result in the occurrence of asthma. Whether GSDME is associated with asthma still requires further research.

PJVK is the most special one among GSDMs, which is derived from the duplication of GSDME. Unlike typical GSDMs, The PJVK is losing the last three exons encoding the CTD and its CTD contains a zinc-finger domain whose function remains unclarified (21, 88). Meanwhile, PJVK lacks the linker region containing protease cleavage sites and cannot be activated by classical protease cleavage (21). So far, no research has confirmed that PJVK has the activity of forming membrane pores. Existing studies have indicated that the core functions of PJVK are concentrated in the auditory system, and its mutations can lead to non-syndromic deafness (89). So far, no studies have found any relevant pathways through which it may affect asthma.

## Conclusion and discussion

GSDMs have been considered as key players in asthma, especially GSDMA, GSDMB, and GSDMD. There are many pathways that have been confirmed to involve in the pathophysiology of asthma. This paper explores the pathophysiological role of GSDMs in asthma and provides an overview of the GSDMs associated with asthma, particularly focusing on GSDMs-mediated pyroptosis, which can cause cell dysfunction, pulmonary fibrosis, and pro-inflammatory cytokine release, ultimately promoting airway inflammation, mucus hypersecretion, airway remodeling, and AHR. These are key pathological features of asthma (74). Except for PJVK, all GSDMs can induce pyroptosis, but the expression of these GSDMs in the lungs and their roles in asthma still need to be further targetedly researched. In addition, recent findings indicate a definite genetic link between GSDMB and asthma. As the GSDM protein with the highest correlation to asthma currently, GSDMB can also affect asthma via pyroptosis and other signaling pathways. While the association of GSDMA is more complex, some studies suggest a link to asthma susceptibility; however, the specific mechanism and strength of this connection still need further research (39). Considering the complexity and diversity of the relationship between GSDMs and asthma, the primary focus of this discussion is the involvement of GSDMs in asthma. In fact, asthma-related cytokines can effectively regulate the expression of GSDMA and GSDMB in *human* bronchial epithelial cells. Th2-related factors (e.g., IL-5), non-Th2-related factors (e.g., IFN-*γ* and TGF-β1), and factors that can be either Th2 or non-Th2 (e.g., LIGHT) can all upregulate the expression of Gasdermins (90). These findings suggest that the Gasdermin family has a close interaction with the cytokine network in the pathogenesis of asthma. Further exploration of the precise regulatory mechanisms and functional roles of GSDMA and GSDMB in different asthma endotypes will not only deepen our understanding of asthma pathogenesis, but also provide potential targets for the development of treatment strategies for asthma.

The current relevant therapies mainly focus on GSDMB and GSDMD. For instance, Wang et al. used artificial intelligence to screen out the GSDMD-NT pore blocker SK56. In the experiment, a single intravenous injection of 1 mg/kg SK56 into the thigh vein of *mice* could selectively block GSDMD-NT pores, delay pyroptosis, thereby inhibiting the release of IL-1β and alleviating inflammation. Moreover, it was demonstrated that this polypeptide can maintain stable activity at 37 °C (*human* body temperature) (91). This makes SK56 a promising treatment option for asthma. Chemically inhibiting cGAS with G140, STING with H151, or TBK1 with BX795 can significantly alleviate the enhanced expression of ISGs and the IFN response induced by GSDMB, and reduce bronchial epithelial damage, providing a new perspective for GSDMB-targeted therapy for asthma (57). Furthermore, the traditional Chinese medicine Tuo-Min-Ding-Chuan Decoction (TMDCD) exerts anti-asthmatic effects on allergic asthmatic *mice* by acting on TLR4-NLRP3 pathway-mediated pyroptosis, providing a basis for the combined treatment of asthma with both traditional Chinese and Western medicine. Mechanistically, TMDCD inhibits the activation of the TLR4-NLRP3 signaling cascade, suppresses Caspase-1-dependent cleavage of GSDMD and subsequent pyroptosis, thereby blocking the release of pro-inflammatory cytokines (IL-1β, IL-18) and alleviating bronchial epithelial injury (92).

In summary, targeted regulation of GSDMs and their related pathways represents a highly promising and precise therapeutic approach for asthma. We may explore the following aspects: first, GWAS have confirmed that genetic variations in the GSDM family genes, especially GSDMB, are significantly associated with the risk of asthma, and their specific SNPs and expression levels are expected to become potential biomarkers for the diagnosis, risk prediction and prognosis evaluation of asthma (93). Second, the GSDMs-mediated pyroptosis pathway plays a key role in airway inflammation and tissue injury, providing an important intervention target for asthma. AI-based target validation, drug screening and molecular design can provide a new paradigm for developing highly selective GSDM family inhibitors. Third, although the currently widely used ICS therapy in clinical practice can effectively control airway inflammation and reduce AHR, long-term and high-dose use may cause systemic adverse reactions (94). Traditional Chinese medicine exerts anti-inflammatory effects by regulating the GSDMD-mediated pyroptosis pathway and can reduce the dosage of ICS in clinical practice, providing reliable experimental evidence for the integrative treatment of asthma (92). Although several key issues remain: mechanistically, the specific regulatory networks of GSDMs and their crosstalk with other cell death pathways have not been not fully elucidated; clinically, GSDM-targeted inhibitors are still under development, with their safety and efficacy yet to be verified. However, increasing studies show that inhibiting the GSDM family and related pathways can alleviate airway inflammation, improve the airway environment, and control asthma, while further exploring GSDM-targeted therapies may provide new ideas and potential approaches for future asthma management.
